# Modeling predictors of behavior of American consumers suffering from food intolerances or food allergies

**DOI:** 10.1371/journal.pone.0325607

**Published:** 2025-06-09

**Authors:** Marija Cerjak, Marcin Adam Antoniak, Daniela Šálková, Benedykt Pepliński, Sylwester Białowąs, Željka Mesić

**Affiliations:** 1 University of Zagreb Faculty of Agriculture, Zagreb, Croatia; 2 Poznan University of Economics and Business, Department of Market Research and Services, Poznań, Poland; 3 Czech University of Life Sciences – Prague, Department of Trade and Finance, Kamýcká, Czech Republic; 4 Department of Law and Enterprise Management in Agribusiness, Poznan University of Life Sciences, Poznań, Poland; Yantai Institute of Technology, CHINA

## Abstract

Food intolerances, allergies, and celiac disease cause hypersensitive reactions to certain foods, collectively termed adverse reactions to food. This study aims to identify predictors of purchasing intentions and behaviors among individuals with such reactions, using three behavioral models: the Theory of Planned Behavior, the Health Belief Model, and Protection Motivation Theory. The authors created a new, comprehensive model by integrating these theories, combining their strengths to provide a more robust framework. The survey, conducted via the Forthright platform using the Computer-Assisted Web Interview (CAWI) method, involved 1,088 respondents. This article is the first to explore predictors of consumer intentions and behaviors regarding food products suitable for individuals with intolerances or allergies, offering new insights into consumer decision-making models.

## 1. Introduction

Food intolerances, food allergies, and celiac disease represent atypical, hypersensitive bodily reactions triggered by the ingestion of specific foods, collectively termed adverse reactions to food (ARF) [1,2], as referred to as adverse food reactions or simply food sensitivities [3]. While these terms are frequently used interchangeably, they entail fundamental distinctions. Food allergy, typically directed towards certain proteins, and celiac disease, which involves gluten, result from an immune-mediated abnormal response to specific food components [4]. However, unlike food intolerance, both food allergy and celiac disease have the potential to induce life-threatening reactions, including anaphylaxis in the case of food allergy [5] and inflammation of the small intestinal mucosa with subsequent villous atrophy in the case of celiac disease [6]. Importantly, while food allergy prompts an immune response against food components, celiac disease entails an immune reaction against one’s own body [1]. Conversely, food intolerance represents a non-immune gastrointestinal sensitivity [4]. The symptoms of these conditions often overlap [7], leading to uncertainty regarding their precise origins.

One-fifth of the population in Western countries believe they experience symptomatic reactions to food [8]. However, medical data suggest that food allergies, intolerances, and celiac disease collectively affect approximately 10–20% of the population [4,9,10]. Interestingly, the prevalence of self-reported diagnoses of food intolerances and allergies often exceeds the rates confirmed by medical tests [5]. Consequently, the true prevalence of ARF remains largely unknown [11].

Adverse reactions to food may have genetic origins, but they can also result from the presence of additives such as dyes, preservatives, and hardeners, which may overload the immune system and contribute to the development of other chronic diseases [4]. The most effective way to avoid such reactions is often through a proper diet that excludes problematic ingredients. In recent years, there has been a surge in popularity of food products free from potentially harmful substances. Consequently, the development of such products has accelerated, and food sensitivity has become a notable social trend [12]. However, concerns have been raised about the nutritional adequacy of these specialized products [13]. Research has also focused on deciphering trends related to “free-from” food, which primarily targets gluten or lactose-free products but extends to items devoid of sugar, fat, and other perceived “safe” ingredients [14,15]. Our aim is to better understand this trend by identifying predictors of food intentions and behaviors among individuals with ARF. Moreover, this research employs methodical, structured approaches that can be utilized by other scientists in various studies, not only those related to ARF but also to different aspects of human health.

Although some recent articles have delved into adverse reactions to food [1–3,16], particularly regarding dietary adherence [17–19], to the best of our knowledge, there is a notable gap in the literature concerning predictors of consumer intentions and behaviors related to food products suitable for individuals with food intolerance or allergies. Furthermore, no articles have analyzed these predictors using established theoretical models. Therefore, our objective is twofold: to identify such predictors and to utilize three well-established behavioral models – the Theory of Planned Behavior (TPB), Health Belief Model (HBM), and Protection Motivation Theory (PMT) – to conduct our analysis. Additionally, we aim to construct a comprehensive model that amalgamates these constructs, leveraging their collective strengths to provide a more nuanced understanding of the phenomenon under investigation.

## 2. Theoretical background

**Fig 1:**
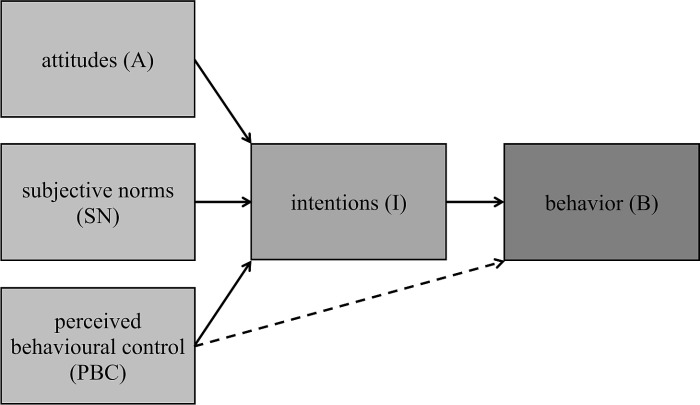
Theory of Planned Behavior model. Note: for consistency, PBC is renamed as PBCSE in the analytical part of this paper.

The Theory of Planned Behavior (TPB) stands as one of the most extensively studied and applied theories for explaining and predicting behavior [20]. According to its originator, Ajzen [21] it ranks among the most widely embraced social-psychological models. The theory posits that human behavioral intentions are shaped by attitudes, subjective norms, and perceived behavioral control [22], with intentions serving as a robust predictor of behavior [23]. In addition, intentions represent the likelihood that a consumer is willing to engage in a specific action [24]. Attitudes, as outlined by Ajzen [25,26] encapsulate an individual’s overall evaluation of a behavior and encompass its positive or negative assessment [27]. The more favorable the attitudes, the greater the likelihood that an individual will engage in the behavior. Subjective norms entail an individual’s perceptions of whether individuals deemed important to them endorse or disapprove of a particular action [25,26]. In essence, they encompass the social pressures or influences that encourage individuals to undertake a specific behavior [28]. Finally, perceived behavioral control, akin to the self-efficacy concept in previously discussed models, centers on an individual’s perception of the ease or difficulty associated with performing a behavior [25,26]. All these factors are depicted in Fig. 1.

**Fig 2:**
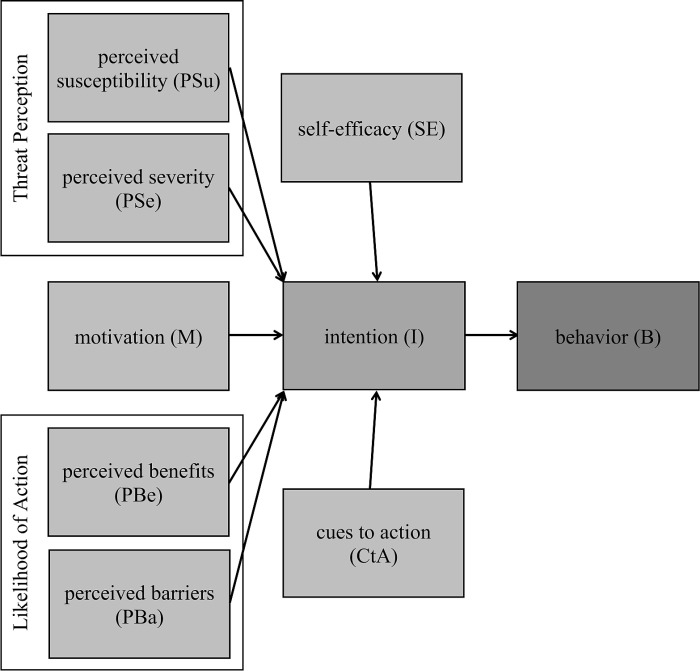
Health Belief Model. Note: for consistency, PBe is renamed as PBRE and SE as PBCSE in the analytical part of this paper.

According to Mahindarathne [29], the Health Belief Model (HBM), depicted in Fig. 2, is one of the most widely used concepts for investigating various health behaviors. Essentially, it serves as a common model for understanding individual differences in coping behaviors related to specific health issues [30]. The HBM is a socio-psychological construct designed to elucidate and predict health-related behaviors [31]. Developed in the 1950s [32] the model aims to raise awareness of the significant health risks associated with preventable diseases [33]. The HBM comprises several key components: perceived vulnerability and severity, collectively known as threat perception; perceived benefits and barriers, which influence the likelihood of action; and health motivation, all of which contribute to determining intentions or behaviors. Furthermore, the model was later expanded to include cues to action and self-efficacy. Perceived susceptibility refers to an individual’s subjective assessment of the risk of a health problem [31,32].

Perceived susceptibility, as outlined by Rosenstock [32], suggests that individuals who perceive themselves as having a low risk of developing a disease are more inclined to engage in unhealthy or risky behaviors. Perceived severity, akin to a variable in the Protection Motivation Theory [32,34], reflects the degree to which individuals perceive health threats as potentially having a negative impact on their lives [35]. Perceived benefits, similar to response efficacy in the Protection Motivation Theory [32,34] pertain to an individual’s assessment of the value or effectiveness of engaging in a particular behavior. Perceived barriers refer to an individual’s evaluation of obstacles hindering behavior change and can diminish the likelihood of engaging in a particular action [31,32]. Health motivation reflects an individual’s desire to act towards achieving a goal. The HBM also posits that some form of trigger, termed “cues to action,” (CtA) is necessary to initiate action [31,32]. Additionally, the model emphasizes self-efficacy, akin to that in the PMT and perceived behavioral control in the TPB, which refers to an individual’s belief in their ability to successfully perform a specific behavior [31,32]. The model also addresses modifying variables, typically demographic or psychological factors.

The Protection Motivation Theory (PMT) was initially developed in 1975 [34] and further refined in 1983 [36,37]. The theory is classified as one of several expectancy-value theories, which suggest that individuals will engage in an action if they anticipate that the expected value will outweigh the effort required for the action [38].

**Fig 3:**
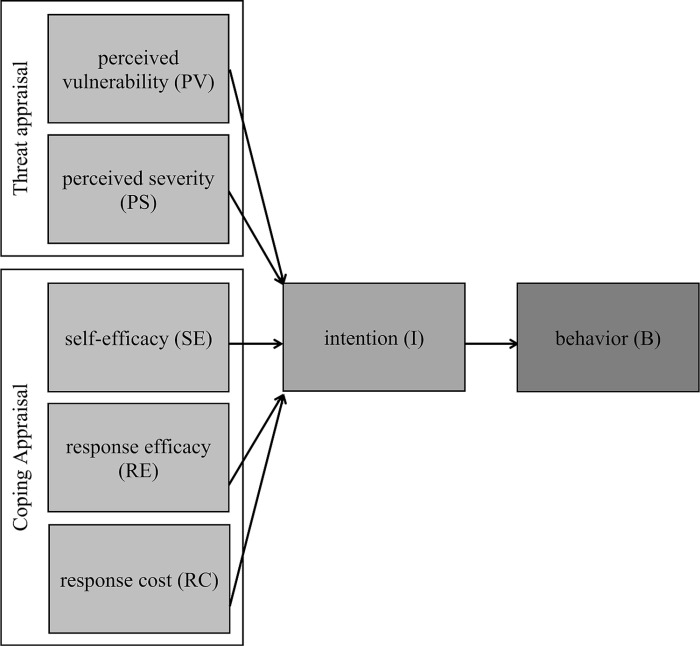
Protection Motivation Theory model. Note: for consistency, RE is renamed as PBRE and SE as PBCSE in the analytical part of this paper.

The strength of PMT lies in its consideration of individuals’ perceptions of threats in terms of severity and susceptibility to experiencing a specific event, as well as the costs associated with avoiding it [39]. The theory is structured around two distinct cognitive processes: threat appraisal and coping appraisal. Threat appraisal pertains to how individuals perceive the severity of and susceptibility to a particular threat, while coping appraisal evaluates various factors that influence individuals to engage in a recommended preventive response [34]. Within threat appraisal, perceived vulnerability refers to an individual’s belief in their susceptibility to a certain threat, such as a disease, while perceived severity, akin to the Health Belief Model, pertains to the belief in the potential harmful effects of the threat. Response efficacy represents the belief that engaging in specific behavior will effectively reduce the threat, similar to the perceived benefits described in the Health Belief Model. Self-efficacy denotes an individual’s belief in their capability to engage in a particular behavior and shares similarities with perceived behavioral control in the Theory of Planned Behavior. Finally, response cost refers to the perceived barriers or obstacles associated with implementing the behavior [34]. PMT is commonly employed in the field of health psychology to explain individuals’ intentions and behaviors concerning health threats and preventive measures across various domains, including adverse reactions to food [40,41]. The PMT model is illustrated in Fig. 3.

According to its creator, TPB is acknowledged as a theory that does not comprehensively elucidate all the predictors ultimately influencing intentions and behavior [25]. This assertion has been corroborated by other researchers who have enhanced model fit by incorporating supplementary constructs [42,43]. However, Noar and Zimmerman [44] contend that both TPB and HBM exhibit deficiencies, as they “do not fully account for the complexity and multidimensionality of behaviors.” For instance, the effect size of primary HBM variables is frequently minimal [45]. Additionally, in the case of HBM, the relationships between variables are not distinctly established [46], a criticism that may also apply to PMT. Wascott [47] asserts that TPB fails to differentiate between factors that may facilitate or inhibit the intention to engage in adaptive behavior, while Sniehotta et al. [48] contend that it is generally a static model. Conversely, PMT has been criticized for not addressing how attitudes may change [49], a critique that extends to HBM, or for its assumption that subjects have not already adopted a coping response [50]. Hence, our objective is to compare these models and devise a new one based on the strengths of their predecessors. The new model is proposed and expounded upon later in the article – Fig. 7.

**Fig 4:**
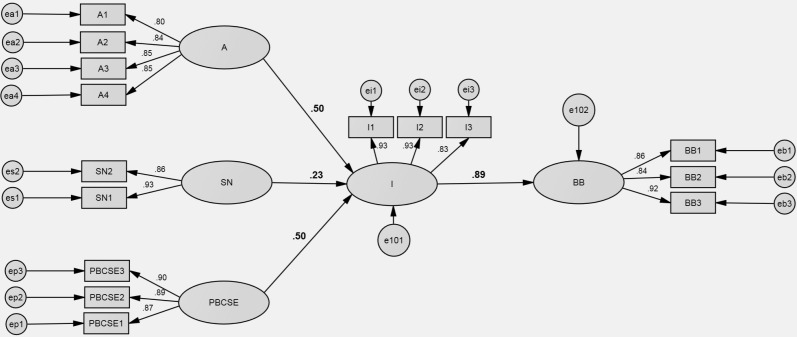
Theory of Planned Behavior model SEM results. Note: A – attitudes, SN – subjective norms, PBSCE – perceived behavioral control, I– intentions, BB – buying behavior.

**Fig 5:**
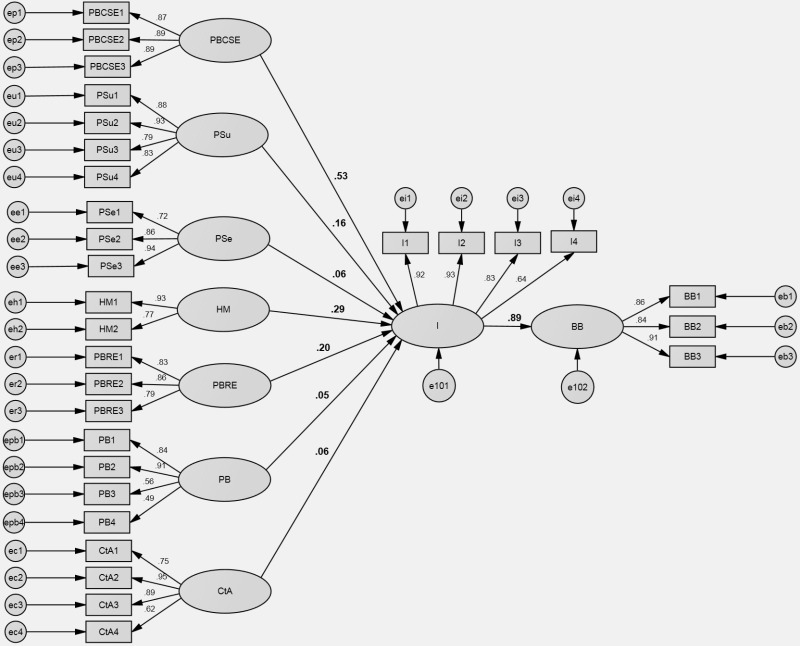
Health Belief Model SEM results. Note: PBCSE – self efficacy, PSu – perceived susceptibility, PSe – perceived severity, HM – health motivation, PBRE – perceived benefits, PB – perceived barriers, CtA – cues to action, I – intentions, BB – buying behavior.

**Fig 6:**
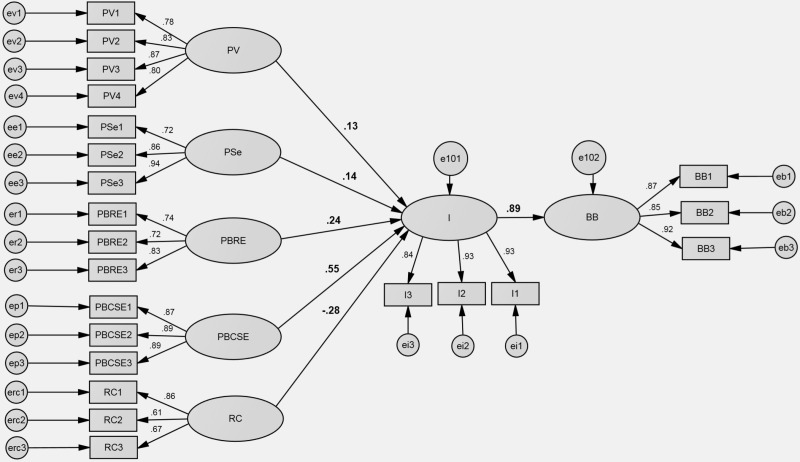
Protection Motivation Theory SEM results. Note: PV – perceived vulnerability, PSe – perceived severity, PBCSE – self efficacy, PBRE – response efficacy, RC – response cost, I – intentions, BB – buying behavior.

**Fig 7:**
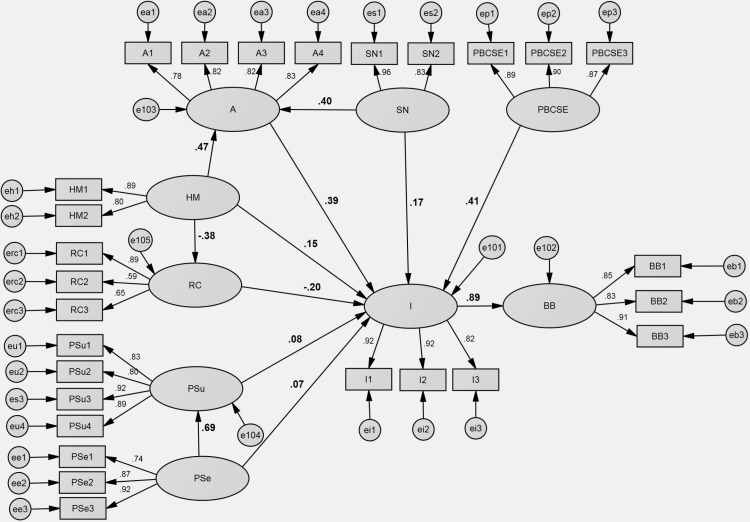
The final (outcome) model SEM results. Note: A – attitudes, SN – subjective norms, PBCSE – perceived behavioral control/ self efficacy, HM – health motivation, RC – response cost, PSu – perceived susceptibility, PSe – perceived severity I – intentions, BB – buying behavior.

## 3. Methods

Our goal was to obtain a final sample of 1,000 respondents. To achieve this, we administered an online questionnaire through the Forthright online panel, beginning with screening questions to identify individuals who were responsible for food purchasing and lived in a household with at least one member affected by a food allergy or intolerance. This screening yielded a refined sample of 1,421 respondents. To further ensure data quality, we included an attention-check question, which resulted in a final sample of 1,088 participants. The inclusion of this check significantly improved the reliability of the survey data.

The demographic breakdown of the sample revealed that 52% were female. Educational attainment varied, with 42% having completed secondary education, 34% holding a bachelor’s degree, and 11% having education beyond a bachelor’s degree. Regarding employment status, 43% reported full-time employment, 11% part-time, 13% retired, 10% unemployed, and 9% self-employed. In terms of racial composition, 69% identified as White non-Hispanic, 14% as Black, and 9% as White Hispanic/Latino, with 63% residing in urban areas. Comparison of the sample structure with the USA population [51] indicates a high degree of representativeness in terms of gender distribution, educational attainment, and racial diversity. More detailed demographic information can be found in Table 1.

**Table 1:** Demographic analysis.

		Frequency	Percent	Valid Percent
**Responsible for food purchases**	Mostly respondent	2928	75.3	75.3
	Respondent and someone else in the household	861	22.2	22.2
	Mostly someone else in the household	97	2.5	2.5
	Total	3886	100	100
**Total analyzed**		**1088**	**100**	
**Respondent or someone from the household suffering from food allergy/intolerance**	Yes	1421	36.6	37.6
	No	2355	60.6	62.4
	Missing	110	2.8	
**Gender**	Female	569	52.3	53.4
	Male	477	43.8	44.7
	Other/ prefer not to say + Missing	20	1.8	1.9
	Missing	22	2.0	
**Age**	18-35	312	28.7	29.3
	36-50	336	30.9	31.5
	51-65	286	26.3	26.8
	66+	132	12.1	12.4
	Missing	22	2.0	
**Education**	Less than a high school diploma	22	2.0	2.1
	High school degree or equivalent	452	41.5	42.4
	Bachelor`s degree	360	33.1	33.8
	Master`s degree	98	9.0	9.2
	Doctorate	21	1.9	2.0
	Other	113	10.4	10.6
	Missing	22	2.0	
**Working status**	Employed full-time	460	42.3	43.2
	Employed part-time	116	10.7	10.9
	Unemployed	103	9.5	9.7
	Student	41	3.8	3.8
	Retired	142	13.1	13.3
	Self-employed	90	8.3	8.5
	Unable to work	113	10.4	10.6
	Missing	23	2.1	
**Race/ ethnical group**	Asian	46	4.2	4.3
	Black	149	13.7	14
	Hispanic/Latino	90	8.3	8.5
	Native	16	1.5	1.5
	White non-Hispanic	729	67	68.5
	Other	27	2.5	2.5
	Prefer not to say	8	0.7	0.8
	Missing	23	2.1	
	Total	1088	100	

The analysis involved three established models rooted in the behavioral economy – the Theory of Planned Behavior, the Health Belief Model, and the Protection Motivation Theory. Each model underwent scrutiny using Structural Equation Modeling (SEM) – a versatile statistical technique utilized to analyze complex relationships among multiple variables within a unified framework [52,53]. This technique can be used to simultaneously examine the measurement and structural aspects of a model, exploring both the relationships between observed variables and latent constructs, as well as the interconnections between these constructs themselves. This method also enables the assessment of direct and indirect effects, moderating relationships, and overall model fit. We have used this method to formulate a composite model aimed at offering a comprehensive insight into the determinants of food purchasing behavior within households where one or more individuals suffer from food allergies or intolerances.

To create the final model, we initially considered all variables derived from the three theoretical frameworks. However, our analysis showed that not all of them were statistically significant. As a result, only those variables that proved to be significant were retained in the final model. This is consistent with both behavioral economics principles and best practices in statistical modeling, where the focus is on parsimony and empirical validity. At the core of our model are intention and buying behavior, which represent the key outcomes of interest. All other constructs function as predictors – factors that influence these two central elements. From this perspective, any variable may be included as long as its relevance is statistically confirmed through confirmatory factor and path analysis. The results, as presented in the Results section, support the soundness of this integrated behavioral model.

During our analysis, we additionally observed that certain variables, despite being labeled differently in the three models, essentially captured similar constructs. Thus, while we retained the original variable names from each model, their abbreviations were chosen to reflect these commonalities across models. The variables utilized in our study included perceived susceptibility (PSu), perceived severity (PSe), perceived vulnerability (PV), health motivation (HM), perceived benefits and response efficacy (PBRE), perceived barriers (PB), response cost (RC), attitudes (A), subjective norms (SN), perceived behavioral control and self-efficacy (PBCSE), cues to action (CtA), intention (I), and buying behavior (BB). These variables were also integrated into the final model developed in our research. Detailed descriptions of these variables are provided in Table 2 of the questionnaire.

**Table 2:** Constructs of the three theories used in the paper.

Variables	Items	Source
**attitudes**	For me buying “free from food” on a regular basis:	[54]
	Is good	
	Pays off	
	Is wise	
	Is useful	
**subjective norms**	My close friends approve of my regular buying of “free from food”	[55]
	My colleagues approve of my regular buying of “free from food”	
**perceived behavioral control (PBC) in TPB + self efficacy (SE) in HBM and PMT (PBCSE)**	I have enough budget to buy “free from food” on a regular basis	[56]
	I feel there is nothing to prevent me from buying “free from food” on a regular basis	
	It is easy for me to buy “free from food” on a regular basis	[57]
**intention (I)**	I intend to buy “free from food” on a regular basis	[55]
	I plan to buy “free from food” on a regular basis	
	I will probably buy “free from food” on a regular basis	
**buying behavior (BB)**	I have been a regular buyer of “free from food”	[58,59]
	I still buy “free from food” even though conventional alternatives are on sale	
	I regularly choose to buy “free from food”	[60]
**perceived susceptibility (PSu)**	In the case of people suffering from adverse reactions to food not following a free from diet may:	[61]
	Threaten their health	
	Increase their psychological worries	
	Deteriorate their health	own
		Reduce the quality of their lives
**perceived severity in HBM and PMT (PSe)**	In the case of people suffering from adverse reactions to food not following a free from diet will:	[61]
	Make them feel bad	
	Have a negative influence on their family life	
	Have a negative influence on their work/ school/ studies	
**health motivation (HM)**	I am motivated to use healthy products for good health	[62]
	I frequently do things to improve my health	own
**perceived benefits (PBe) in HBM and response efficacy (RE) in PMT (PBRE)**	In the case of people suffering from adverse reactions to food sticking to the “free from diet” will help them:	[63]
	Prevent the disease side effects	
	Control the disease	
	Feel better	
**perceived barriers (PBa)**	Following a “free from” diet on a regular basis requires:	[61]
	Spending more time	
	Putting more effort into	
	Spending more money	
	Adopting new habits, which is difficult	own
**cues to action (CtA)**	My family members regularly remind me to buy “free from food”	own
	My close friends regularly remind me to buy of “free from food”	
	My colleagues regularly remind me to buy “free from food”	
	My doctor regularly reminds me to buy “free from food”	
**perceived vulnerability (PV)**	Adverse reactions to food can negatively affect people suffering from this disease	[57]
	People suffering from adverse reactions to food will experience negative effects of this disease in their lifetime	
	People suffering from adverse reactions to food are vulnerable to negative effects of this disease.	
	For people suffering from adverse reactions to food, chances of being negatively affected by this disease are high	
**response cost (RC)**	Although “free from food” is better for my health, I am not willing to pay more for it	[57]
	I usually compare prices of “free from” products, and will only buy the ones when the price is reasonable to me	
	Purchases of “free from food” would require too much of an adjustment in my food consumption	

All analyzes were performed using SPSS 29.0 and AMOS 22. Software packages.

The survey was conducted exclusively with adult respondents aged 18–85 and spanned from September 20, 2023, to December 4, 2023. Informed consent was obtained from participants who agreed to participate by accepting the survey conditions, and this consent was stored accordingly. Additionally, the requirement for explicit consent was waived by the ethics committee. The study received formal approval from the Committee of Ethical Science Research Involving Human Participants at the Poznań University of Economics and Business, Poland (original ethics approval letter – Resolution 6202).

The research employed the Computer-Assisted Web Interviewing (CAWI) method and was administered through the Forthright online panel. The purpose of the study was transparently communicated to participants prior to the commencement of the survey, as detailed in the introductory statement:

*“Dear participant, we would like to ask you to participate in research on food intolerances and allergies. The aim of the research is to assess various purchase behaviors. The research is completely anonymous, voluntary and carried out solely for scientific purposes, so it is very important to provide honest and true answers. The questionnaire will present statements to which we ask you to respond carefully. If for any reason you decide to stop the survey, you can do so at any time without any consequences. In this case, however, your survey will be rejected. If you have read carefully and agree with the above statements, please proceed to the first question”.*

Participants indicated their informed consent by clicking the “Next page” button. They were also compensated financially for their participation.

The authors were not involved in any legal matters associated with the survey process. All responsibilities related to participant recruitment, compensation, and legal compliance were handled solely by the Forthright platform owner.

## 4. Results

Our analytical process comprised a sequential series of assessments aimed at validating iterative models derived from foundational constructs. The culmination of this process was the synthesis and refinement of these models into a final iteration. Methodical scrutiny of individual outcomes was undertaken to discern the most appropriate representation. While numerous analyses were conducted and several interim models emerged, we herein present solely those analyses pertinent to the foundational models, alongside the ultimate selection designated as the final model (also referred to as the outcome or integrated model).

### 4.1. Measurement model testing

First, we evaluated the measurement models [64–66]. We used the confirmatory factor analysis (CFA), which serves as the primary method to assess the measurement model’s validity. By analyzing various outputs from CFA, we could determine the appropriateness of the measurement model and identify any necessary adjustments [67]. One crucial aspect to consider is the standardized factor loadings (referred to as standardized regression weights in CFA). Ideally, factor loadings for all indicators (measured or observed variables in SEM) should exceed 0.5, preferably surpassing 0.7. A low factor loading indicates that the indicator does not sufficiently contribute to explaining the variance. It’s important to explicitly mention the exclusion of each indicator in the text. In addition to fit indices (such as χ2//df, GFI, CFI, RMSEA), assessing construct reliability and validity is crucial. Construct reliability typically involves examining Cronbach’s α (a measure of internal reliability). Construct validity is commonly evaluated using the average variance extracted (AVE), which measures convergent validity. Discriminant validity is assessed by comparing the AVE of each construct with its squared correlation with any other construct in the model; ideally, the AVE of each construct should exceed its squared correlation with any other construct.

The internal consistency of the items used in our study was examined based on the Cronbach’s α parameter. Scores ranged from 0.750 to 0.942 – above the minimum acceptance level of 0.6 [68]. Convergent validity was confirmed on the basis of the Composite Reliability (CR) and average variance extracted (AVE). All parameters met the minimum requirements – CR > 0.7 and AVE > 0. 5 [69,70] as can be seen in Table 3.

**Table 3:** Convergent validity of the constructs.

	CR (composite reliability)	AVE (average variance extracted)
**I**	0.945	0.853
**BB**	0.926	0.806
**SN**	0.888	0.799
**A**	0.898	0.689
**PSu**	0.903	0.758
**PSe**	0.882	0.716
**HM**	0.835	0.717
**RC**	0.760	0.520
**PBCSE**	0.916	0.785

For the structural equation modeling (SEM) analysis, we utilized full information maximum likelihood estimation (FIML). FIML is a robust statistical method that accommodates missing or incomplete data, allowing for accurate parameter estimates and model fit evaluation. By incorporating all available data, FIML enhances statistical power, reduces bias, and improves the precision of model parameter estimation. This approach ensures that the SEM model relies on complete information, resulting in more reliable and valid results [71].

The Theory of Planned Behavior was initially analyzed, and the measurement model exhibited a high level of quality. The Chi-Square value is substantial and significant (χ^2^ = 269.538; df = 80). Considering the sample size, the χ^2^/df ratio falls below 5 (3.369), indicating reasonable fit. The Comparative Fit Index (CFI) and Tucker-Lewis Index (TLI) values of 0.987 and 0.981 respectively, suggest a robust model fit. Moreover, the Root Mean Square Error of Approximation (RMSEA) value of 0.047 with a 90% confidence interval ranging from 0.041 to 0.053 further affirms the adequacy of the measurement model.

Regarding the Health Belief Model, it also exhibits a good fit with the following values: χ^2^ = 1343.346; df = 369 and χ^2^/DF = 3.641. The Comparative Fit Index (CFI) and Tucker-Lewis Index (TLI) are.963 and.954 respectively. The Root Mean Square Error of Approximation (RMSEA) is.043 with a 90% confidence interval between.041 and.046.

For the Protection Motivation Theory, the measurement model also demonstrates a good fit with the following values: χ^2^ = 674.600; df = 188 and χ^2^/df = 3.588. The Comparative Fit Index (CFI) and Tucker-Lewis Index (TLI) are.973 and.964 respectively. The RMSEA is.043, with a 90% confidence interval between.039 and.046.

In the analysis of the integrated model, the χ^2^ is high and significant (χ^2^ = 811.788; df = 288). Considering the sample size, the χ^2^/df ratio is 2.819 which falls below the threshold of 5, indicating a reasonable fit. The Comparative Fit Index (CFI) and Tucker-Lewis Index (TLI) values of 0.978 and 0.971 respectively, indicate a strong model fit. Additionally, the Root Mean Square Error of Approximation (RMSEA) value of 0.041 with a 90% confidence interval ranging from 0.038 to 0.044 further supports the adequacy of the measurement model.

### 4.2. Composite model

Based on the analysis conducted using Structural Equation Modeling (SEM) – path analysis, the model based on the Theory of Planned Behavior exhibits a poor fit, as indicated by the following statistics: χ^2^: 857.082; df: 86; χ^2^/df: 9.966. The Comparative Fit Index (CFI) and Tucker-Lewis Index (TLI) values are 0.951 and 0.931 respectively. Additionally, the Root Mean Square Error of Approximation (RMSEA) value is 0.079 with a 90% confidence interval ranging from 0.075 to 0.084.

The analysis reveals that attitudes (A) and perceived behavioral control (self-efficacy in the following two models) (PBCSE) exert a strong influence on intentions (I). There is a slightly weaker relationship observed between subjective norms (SN) and intentions (I). The path coefficients further elucidate the strength of these relationships, underscoring the significant impact of attitudes on shaping intentions and, subsequently, their influence on purchasing behavior (BB). These relationships together with Beta (β) parameters are visually represented in Fig. 4.

The SEM analysis confirms that the Health Belief Model (path model) can be accepted, as indicated by the fit statistics (χ^2^: 3222.135; df: 397; χ^2^/df: 8.116). The CFI and TLI values of 0.888 and 0.869 respectively, demonstrate a poor fit. Additionally, the RMSEA value of 0.081 with a 90% confidence interval ranging from 0.078 to 0.083. The relations in the model are statistically significant. The standardized regression weights indicate the strength and direction of the relationships between the constructs in the Health Belief Model (HBM).

The analysis underscores several significant relationships among the variables. Notably, there is a strong positive association between intentions (I) and buying behavior (BB) indicating that individuals with stronger intentions are more likely to engage in actual buying behavior related to health-related products for individuals with food allergies or intolerances. Furthermore, intentions exhibit robust positive relationships with health motivation (HM) and self-efficacy (perceived behavioral control in the TPB model) (PBCSE). This suggests that individuals who are highly motivated to prioritize health and perceive themselves as capable of effectively engaging in health-related behaviors are more likely to intend to purchase suitable food products. Additionally, perceived barriers (response efficacy in the Protection Motivation Theory) (PBRE) and perceived susceptibility (PSu) positively influence intentions, indicating that individuals who perceive the benefits of adopting health-related behaviors and perceive themselves as susceptible to adverse health outcomes are more inclined to intend to purchase such products. However, perceived severity (PSe) perceived barriers (PB) cues to action (CtA) demonstrate a weaker influence on intentions, implying that these predecessors may enhance individuals’ intentions to buy health-related products. The findings, in conjunction with the Beta (β) parameters illustrated in Fig. 5, underscore the significance of targeting self-efficacy, perceived susceptibility, health motivation, and perceived benefits to foster buying behavior among individuals dealing with food allergies or intolerances.

The model based on the Protection Motivation Theory (PMT) has a poor fit, as indicated by the structural equation modeling (SEM) parameters: χ2 value of 17249.310 with df = 203 and a χ2/df ratio of 8.617. Additionally, the CFI and TLI values of.914 and.893, respectively, fall below the recommended threshold of.95. Furthermore, the RMSEA value of.084, with a 90% confidence interval from.080 to.087 also suggests a poor fit. The relations in the model are statistically significant.

Despite the poor fit of the model, there are still notable relationships observed within it. One significant finding is the very strong positive relationship between intention (I) and self-efficacy (PBCSE), indicating that individuals’ intentions to engage in buying behavior are strongly influenced by their perceived behavioral control. Additionally, intention is strongly related to response efficacy (PBRE), suggesting that perceived benefits play a significant role in shaping individuals’ intentions to engage in buying behavior. Furthermore, intention shows moderate but still positive relationships with perceived severity (PSe) and perceived vulnerability (PV), indicating that individuals’ intentions to engage in buying behavior are influenced by their perceptions of the seriousness of potential health issues and their susceptibility to them. However, intention is negatively related to response costs (RC), implying that perceived barriers may hinder individuals’ intentions to engage in health-related behaviors, such as buying food products suitable for individuals with food allergies or intolerances. While these relationships provide valuable insights into the factors influencing individuals’ intentions and behaviors, the poor fit of the model suggests that it may not accurately capture the underlying data. The SEM analysis for the Protection Motivation Theory is presented below in Fig. 6.

The structural equation modeling – path analysis conducted for the final, outcome model – which integrates the previous three models, demonstrates a significantly improved fit compared to earlier iterations. The fit statistics confirm the adequacy of the model, with a χ2 of 1753.231 and 312 degrees of freedom, resulting in a χ2/df ratio of 5.619. The Comparative Fit Index (CFI) and Tucker-Lewis Index (TLI) values of 0.939 and 0.926 respectively, indicate a strong fit. Furthermore, the Root Mean Square Error of Approximation (RMSEA) value of 0.065, with a 90% confidence interval ranging from 0.062 to 0.068 further supports the adequacy of the model. While some researchers advocate for stricter cutoffs, it is important to note that these serve as guidelines rather than absolute rules. As emphasized by Hair et al. [72], model modifications should be theoretically justified rather than driven by data alone. Our current model represents a theoretically sound integration of established behavioral frameworks while maintaining an acceptable statistical fit. Overall, the fit indices suggest that the final integrated model provides a satisfactory representation of the data and offers valuable insights into the predictors of consumers’ intentions and behaviors regarding food products suitable for individuals with food allergies or intolerances. The relationships within the model are statistically significant.

To systematically evaluate the improvements offered by our integrated model compared to the individual theoretical models, we have included a Table 4 presenting the comparative statistics across all models.

**Table 4:** Comparative statistics of original and integrated models.

Model	χ²/df	CFI	TLI	RMSEA (90% CI)	AIC	Squared Multiple Correlations
						**Intention (I)**
TPB	9.966	0.951	0.931	0.079 (0.075-0.084)	955.082	0.554
HBM	8.116	0.888	0.869	0.081 (0.078-0.083)	3418.135	0.445
PMT	8.617	0.914	0.893	0.084 (0.080-0.087)	1893.310	0.478
Integrated Model	5.619	0.939	0.926	0.065 (0.062-0.068)	1939.231	0.591

The integrated model demonstrates substantial improvement in explanatory power for intention compared to any of the individual theoretical models. When comparing Squared Multiple Correlations (SMC, which are equivalent to R² in regression analysis), our integrated model explains 59.1% of the variance in intention, which is an improvement of 3.7 percentage points over the TPB model (ΔSMC = 0.037), 14.6 percentage points over the HBM model (ΔSMC = 0.146) and 11.3 percentage points over the PMT model (ΔSMC = 0.113).

While all models explain comparable amounts of variance in behavior (ranging from 78.5% to 79.8%), the integrated model achieves this with a substantially improved fit for the RMSEA index (0.065), which is significantly better than any of the individual models (ranging from 0.079 to 0.084). This demonstrates that our integrated model achieves better parsimony while maintaining strong explanatory power for behavioral outcomes.

Furthermore, while TPB shows the best individual model fit for CFI and TLI, it does so with a much simpler model structure. The integrated model maintains strong fit indices while incorporating a more comprehensive theoretical framework, as evidenced by its balanced performance across all fit metrics.

The comparison of AIC values must be interpreted with caution in this case, as the models differ substantially in complexity and number of variables. However, the relatively modest increase in AIC for our integrated model compared to TPB, despite its much greater complexity, further supports the value of our integrated approach.

These results provide robust quantitative evidence that our integrated model offers significantly greater explanatory power for intention – the key theoretical construct linking predictors to behavior – while maintaining good model fit. This supports our argument that combining elements from these established behavioral theories provides a more comprehensive framework for understanding consumer behavior related to food allergies and intolerances.”

In addition, the outcome model provides a comprehensive understanding of the complex interplay of factors influencing health-related behaviors, particularly in the context of food purchasing decisions in households with food allergy or intolerance sufferers. Some variables were excluded from the model due to their lack of significance in the more complex situation. Specifically, the excluded variables are: perceived vulnerability (PV), perceived benefits response efficacy (PBRE), perceived barriers (PB) and cues to action (CtA). The relations stated in the previous models were confirmed.

The analysis indicates that perceived behavioral control/self-efficacy (PBCSE) and attitude (A) have the strongest impact on intention (I) to buy food products suitable for individuals with food allergies or intolerances. Additionally, health motivation (HM) and subjective norms (SN) exhibit a moderate impact on intentions. Perceived severity (PSe) and perceived susceptibility (PSu) also have a statistically significant, albeit low, impact on intention. Interestingly, response cost (RC) shows a moderate but negative impact on intention, suggesting that perceived barriers or obstacles associated with implementing the behavior may hinder individuals’ intentions to purchase relevant products for individuals with food allergies or intolerances, including celiac disease.

Moreover, noteworthy are the additional connections between the variables. Perceived susceptibility serves as a strong moderating construct between perceived severity and intention, indicating its significant role in influencing intention based on perceived susceptibility to food-related issues. Additionally, response cost and attitudes act as moderating constructs for the relationship between health motivations and intention, indicating their role in influencing intention based on health motivation.

All the above analysis and the outcome model are shown in Table 5 and Fig. 7.

**Table 5:** Variables reduction – final model sample analysis.

Variables/ items			All-in-one outcome model					Final model				
			Estimate	Estimate (standardized) – β	S.E.	C.R.	P	Estimate	Estimate (standardized) – β	S.E.	C.R.	P
A	<---	SN						0.296	0.398	0.024	12.168	***
PSu	<---	PSe						0.575	0.693	0.025	22.588	***
A	<---	HM						0.480	0.474	0.034	14.168	***
RC	<---	HM						−0.434	−0.376	0.046	−9.493	***
I	<---	PSu	0.074	0.086	0.018	3.996	***	0.068	0.076	0.030	2.260	0.024
I	<---	PSe	0.058	0.082	0.015	3.809	***	0.054	0.072	0.025	2.120	0.034
I	<---	HM	0.234	0.208	0.025	9.280	***	0.167	0.148	0.036	4.659	***
I	<---	PBRE	−0.026	−0.026	0.023	−1.139	0.255					
I	<---	PB	0.113	0.073	0.034	3.314	***					
I	<---	CtA	0.037	0.041	0.019	1.912	0.056					
I	<---	RC	−0.194	−0.234	0.020	−9.653	***	−0.193	−0.198	0.027	−7.124	***
I	<---	PBCSE	0.293	0.442	0.015	19.460	***	0.294	0.412	0.017	17.160	***
I	<---	A	0.392	0.409	0.023	17.334	***	0.437	0.394	0.037	11.759	***
I	<---	SN	0.159	0.205	0.019	8.340	***	0.140	0.169	0.023	6.192	***
BB	<---	I	0.964	0.884	0.026	36.790	***	0.962	0.886	0.029	33.695	***
PSu1	<---	PSu	1.000	0.830				1.000	0.827			
PSu2	<---	PSu	0.967	0.792	0.028	35.112	***	0.980	0.799	0.032	30.960	***
PSu3	<---	PSu	1.137	0.929	0.026	44.489	***	1.133	0.923	0.029	38.448	***
PSu4	<---	PSu	1.085	0.882	0.026	41.420	***	1.096	0.887	0.030	36.298	***
PSe1	<---	PSe	0.714	0.724	0.023	31.672	***	1.000	0.919			
PSe2	<---	PSe	0.929	0.857	0.024	38.827	***	0.958	0.866	0.025	37.576	***
PSe3	<---	PSe	1.000	0.938				0.748	0.743	0.025	29.720	***
HM0	<---	HM	0.843	0.647	0.035	24.338	***					
HM1	<---	HM	1.152	0.931	0.042	27.393	***	1.069	0.895	0.051	20.945	***
HM2	<---	HM	1.000	0.769				1.000	0.796			
PBRE1	<---	PBRE	0.693	0.739	0.029	24.060	***					
PBRE2	<---	PBRE	0.849	0.720	0.036	23.801	***					
PBRE3	<---	PBRE	1.000	0.833								
PB1	<---	PB	1.726	0.836	0.082	21.120	***					
PB2	<---	PB	1.749	0.918	0.087	20.210	***					
PB3	<---	PB	1.000	0.554								
CtA1	<---	CtA	1.487	0.943	0.059	25.397	***					
CtA2	<---	CtA	1.421	0.896	0.055	25.853	***					
CtA3	<---	CtA	1.000	0.615								
RC1	<---	RC	1.006	0.735	0.044	22.637	***	1.386	0.887	0.080	17.333	***
RC2	<---	RC	0.866	0.636	0.042	20.459	***	0.918	0.591	0.056	16.298	***
RC3	<---	RC	1.000	0.745				1.000	0.653			
A1	<---	A	1.000	0.796				1.000	0.785			
A2	<---	A	1.062	0.837	0.031	34.605	***	1.066	0.823	0.037	28.767	***
A3	<---	A	0.966	0.849	0.027	35.218	***	0.940	0.820	0.033	28.626	***
A4	<---	A	0.955	0.851	0.027	35.271	***	0.948	0.830	0.033	29.020	***
SN1	<---	SN	1.000	0.943				1.000	0.956			
SN2	<---	SN	0.934	0.841	0.071	13.185	***	0.919	0.833	0.044	20.849	***
PBCSE1	<---	PBCSE	0.985	0.870	0.022	45.110	***	0.986	0.867	0.025	39.091	***
PBCSE2	<---	PBCSE	1.065	0.892	0.023	46.888	***	1.071	0.896	0.026	41.064	***
PBCSE3	<---	PBCSE	1.000	0.896				1.000	0.894			
I1	<---	I	1.000	0.907				1.000	0.921			
I2	<---	I	1.020	0.908	0.019	52.731	***	1.019	0.923	0.020	51.320	***
I3	<---	I	0.997	0.800	0.025	40.488	***	1.000	0.824	0.026	38.923	***
BB1	<---	BB	1.000	0.848				1.000	0.854			
BB2	<---	BB	0.992	0.828	0.026	38.271	***	0.992	0.835	0.029	34.520	***
BB3	<---	BB	1.035	0.906	0.024	43.945	***	1.035	0.910	0.026	39.705	***

## 5. Discussion

Health concerns have become a prominent driver shaping food consumption patterns in industrialized societies [72]. This trend is greatly fueled by the escalating prevalence of diet- and lifestyle-related ailments [73,74]. Moreover, there has been a notable rise in the occurrence of allergies and intolerances to certain foods or ingredients [75,76]. This expanding population grappling with food intolerances, allergies, and conditions like coeliac disease has led to the emergence of a market for products devoid of specific ingredients known to trigger adverse reactions to food (ARF), such as gluten and lactose [4,10,12].

Following this trend, the objective of this study was to delve into consumer intentions and behavior, alongside investigating the determinants influencing such intentions and behavior concerning food products specifically designed for individuals with food allergies or intolerances. To accomplish this, we examined and compared three well-established behavioral theories: the Theory of Planned Behavior (TPB), Health Belief Model (HBM), and Protection Motivation Theory (PMT). Each model offers unique insights into the determinants of health behaviors related to food allergies and intolerances. Subsequently, we introduced a novel integrated model, also termed the outcome model, by synthesizing these three theories. We successfully developed a construct that amalgamates the strengths of the previous theories, offering a more nuanced understanding of consumer intentions and their antecedents in the context of the products under study. This methodological advancement significant progress in our research, furnishing a holistic framework for comprehending consumer decision-making in this domain.

### 5.1. The outcome model and determinant of consumer’s intentions and buying behavior

We sought to clarify how the aforementioned theories predict consumers’ intentions and behaviors when purchasing foods suitable for individuals with food intolerances or allergies. By scrutinizing these models, our aim was to offer insights into the intricate consumer decision-making processes concerning food purchases for individuals grappling with food intolerances or allergies.

#### 5.1.1. The analysis of the impact of chosen variables on intentions in the outcome model.

Despite the Theory of Planned Behavior (TPB) model showing poor fit in our study, variables associated with TPB significantly influenced intentions in the final model. Particularly noteworthy is the substantial impact of perceived behavioral control/self-efficacy (PBCSE). This variable emerged consistently across the three base models, exerting a significant influence on intentions, as evident in the final model (ß = 0.41). Numerous studies have demonstrated that heightened perceived behavioral control correlates with a stronger intention to engage in specific behaviors [77,78]. Our study corroborates prior research, such as Shin and Hancer’s [79], which elucidated the influence of perceived behavioral control on purchase intentions in the context of food. Thus, we can conclude that consumers, when confident in their ability to purchase and consume products suitable for their health conditions, are primarily guided by this assurance – an aspect likely significant in highly individualized societies like the United States.

A similarly strong influence was noted for attitudes (A), which also significantly impacted intentions in the TPB. The strong influence of attitudes on intentions has been demonstrated in other studies [43,80]. Thus, not only self-efficacy but also personal attitudes are influential factors shaping intentions and behavior in purchasing products safe for individuals with allergies and intolerances.

To a slightly lesser extent, subjective norms (SN) influenced intentions. This finding is consistent with results obtained by Hagger et al. [81] and Wang & Chou [82] suggesting that in individualized societies, the opinions of others in critical aspects such as health may not be as important (though still relevant) as attitudes and PBCSE.

Variables from the other two models, except for PBCSE, had a lesser impact on intentions. However, health motivation (HM), which appears in the Health Belief Model (HBM), emerged as a significant factor in our study, consistent with other research [83,84]. In the context of potentially harmful ARF, the significant impact of HM on intentions is rational. Our model aligns with previous research indicating that health motivation is a key factor in food choices [85,86].

Perceived severity (PSe) and perceived susceptibility (PSu) exhibited low significance. Interestingly, perceived susceptibility to negative health effects or the perceived magnitude of harm from certain substances does not exert as great an influence on consumer intentions and behaviors as attitudes or PBCSE. The low impact of these variables on intentions has been noted in other studies [87].

Finally, an interesting relationship was observed between response cost (RC) and intentions. Both in our model and in HBM, this influence was negative, consistent with studies by Feng et al. [88] and Schacherer & Hazeltine [89] but inconsistent with findings by Al Jarah & Emeagwali [90] and Keeney et al. [91]. A negative response cost suggests that individuals perceive the obstacles or sacrifices associated with intentions to adopt a particular health behavior to be significant barriers, leading to reduced motivation to engage in that behavior. Similar results were obtained by Schwarzer & Luszczynska [92], and Stillman & Woolley [93] indicating a lower willingness to purchase products healthy for them when they are less available, more expensive, perceived as less palatable or containing too many undesirable ingredients [94,95]. This issue certainly warrants further investigation in the context of various health conditions where diet plays a significant role.

Our findings indicated a significant negative impact of response costs (price barriers) on consumers’ intentions to purchase food suitable for people with food intolerances or allergies. Consumers often perceive higher prices for specialty foods as a loss rather than a mere cost, which significantly influences their purchasing behavior [96]. This perception is based on loss aversion, where the emotional impact of a loss is felt more strongly than the pleasure of an equivalent gain [97]. A study identified that approximately 28% of consumers are significantly affected by rising food prices, often leading to changes in their purchasing habits due to financial constraints [98]. These consumers tend to reflect pessimism and lower life satisfaction, which may increase their sensitivity to price rises and further discourage the purchase of specialty foods [99].

Although loss aversion plays a major role in consumer behavior in relation to specialty foods, it should be taken into account that not all consumers react in the same way [100]. Socioeconomic status and individual values can significantly influence purchasing decisions, suggesting a complex interplay between loss perception and consumer behavior [101]. The perception of specialized food products as too expensive is often based on established reference prices associated with mainstream products [102]. Reference prices serve as benchmarks against which consumers assess the value of products and significantly influence their purchasing decisions [103]. When prices of specialized products are higher than these reference points, consumers may experience cognitive dissonance, leading to a negative assessment of the product [104].

#### 5.1.2. Moderator analysis in the outcome model.

Moderation analysis in statistical modeling examines how one variable influences the relationship between two other variables. In our study, we observed several significant moderations:

- Subjective norms (SN) significantly influenced intentions indirectly through shaping attitudes (A) (β = 0.40). Thus, even though SN had limited direct effects, it played an important role indirectly.
- Health motivation (HM) not only directly influenced intentions (I) but also moderated:
  - the relationship between attitudes (A) and intentions (I) (β = 0.47).
  - the relationship between response cost (RC) and intentions (I) (β = -0.38).
  - When health motivation was low, even positive attitudes or relatively lower perceived costs did not necessarily lead to stronger intentions to purchase allergy-friendly foods.
- Perceived severity (PSe) strongly moderated the relationship between perceived susceptibility (PSu) and intentions (I) (β = 0.69). Thus, the impact of perceived susceptibility was particularly dependent on how severe individuals perceived potential negative consequences.

While the direct impact of subjective norms (SN) on intention was not substantial, SN appears to shape attitudes, which subsequently influence intention [105]. Similarly, health motivation (HM) not only directly impacts intentions but also moderates the relationships between attitudes (A) and intentions (I), as well as between response cost (RC) and intentions [106,107]. Further analysis of HM’s moderation effects in future studies is warranted.

Both attitudes and perceived response cost depend on health motivations. If health motivations are low, intentions to purchase products suitable for individuals with ARF may also be low, despite positive attitudes toward dietary adherence or a relatively higher perceived cost associated with non-compliance [94,108]. Interestingly, perceived severity (PSe) acts as a moderator between perceived susceptibility (PSu) and intentions, despite the small direct effects of both factors on intentions, highlighting the significant moderation effect in this case. Consumers may risk non-compliance with a specific diet if they do not perceive the severity of the side effects of non-compliance with the prescribed dietary regimen. This finding suggests that the influence of PSu may vary depending on the perceived severity of the adverse effects.

The primary contribution of this study lies in the development of a unified model that integrates the strengths of previous frameworks, providing a nuanced understanding of the multifaceted factors influencing health behaviors, particularly regarding food purchasing decisions in households affected by food allergies or intolerances. By combining these established theories, the study expands our theoretical understanding and provides valuable insights for researchers, policymakers, and practitioners seeking to address the complex challenges associated with food allergies and intolerances.

#### 5.1.3. Reflection on cultural differences.

Understanding consumer behavior in relation to food intolerances and allergies requires a careful examination of the cultural context [109]. Cultural factors significantly influence eating habits and impact on how individuals perceive and respond to food-related issues [110]. This interaction between culture and consumer behavior is essential for developing effective strategies to address food intolerances and allergies [111].

Our study focuses on American consumers who often exhibit individualistic attitudes toward health and eating, emphasizing personal responsibility, self-efficacy, and individual decision-making regarding health behaviors. In collectivistic societies, dietary choices and adherence to health routines are significantly influenced by subjective norms, family dynamics and social expectations [112]. This phenomenon is reflected in various studies that examine the correlation between collectivism and consumer decision-making [113,114]. These cultural contexts shape individual behavior and often lead to adherence to group norms and shared dietary practices [115]. Individuals in collectivistic cultures often conform their eating behavior to that of their social groups, as conformity is considered adaptive and beneficial [116]. Research suggests that perceived social norms strongly influence dietary intentions, with individuals more likely to follow a healthy diet if they believe their social environment values such choices [117,118]. What is more, the family plays a crucial role in shaping eating habits, as collective family norms can determine dietary preferences and health behavior [119]. Therefore, replicating this research in collectivistic cultural contexts could reveal important differences in the prediction of consumer intentions and behaviors. Such studies would not only help validate the integrated behavioral model proposed in this article but may also highlight the need for adjustments to account for culturally specific variables.

### 5.2. Managerial implications for companies and marketers

Understanding consumer intentions is crucial for marketing and product design in the sector of products aimed at individuals suffering from ARF [120]. Researching these intentions aids in developing targeted strategies to meet consumer needs and preferences. Manufacturers and retailers should integrate these insights when designing communication and advertising campaigns to effectively engage their target audiences. This approach can enhance consumer awareness and acceptance of products tailored to individuals with food intolerances and allergies. Marketing strategies that emphasize the unique features and benefits of foods free from substances harmful to individuals suffering from ARF could significantly boost sales [121]. With the growing interest in healthy lifestyles and health-related issues, there is substantial market potential for these products [122].

Educating consumers about the benefits of foods free from substances harmful to them is crucial for expanding the market share of such products. A comprehensive approach to consumer education and support is essential for aiding families with food allergies or intolerances and encouraging healthier shopping habits. Factors influencing the purchasing decisions of households with allergies include the perceived effectiveness of these products in preventing negative health effects from consuming harmful substances [11]. Individuals’ perceived susceptibility to adverse reactions positively impacts their willingness to engage in health-oriented activities [123]. Increased awareness facilitates informed decision-making and promotes interest in products with clearly labeled ingredients [124]. Consequently, the demand for products suitable for people suffering from ARF is increasing and driving market expansion [125].

### 5.3. Other implications

Our model and its application extend to scientific, legal, and health policy domains. Adverse reactions to food are prevalent in primary healthcare and have significant physical, psychological, and social consequences. The diverse causes and symptoms often perplex clinicians, resulting in undifferentiated testing and counseling [126]. Inadequate consumer management of food allergies or intolerances, including non-compliance with recommended diets, can exacerbate these conditions to potentially life-threatening levels [2,127]. Such mismanagement disrupts normal lifestyle habits and imposes a substantial economic burden due to the search for alternative dietary options [128]. Individuals of all ages with allergies and intolerances suffer from nutritional deficiencies, anxiety, and social isolation [129].

To enhance our understanding of consumer behavior concerning foods suitable for individuals with intolerances or allergies and to identify the determinants of consumer intentions, it is essential to adapt to evolving consumer demands through transparent food labeling. This approach can significantly benefit public health [130,131] and encourage legislators to consider appropriate regulations. Ultimately, our model, which integrates three established behavioral models, offers a comprehensive approach to understanding consumer intentions and behaviors. It serves as a valuable tool for researchers exploring these areas.

### 5.4. Limitations and direction for future research

Our study investigated consumer behavior and purchase intentions in relation to foods that are free from allergens or intolerances. Despite valuable findings, our study has limitations. It is geographically limited, potentially affecting generalizability, and was conducted in the highly individualistic United States. Future research should examine the model in different cultural and geographic contexts.

Because the focus is on a specific customer group and health-related products, the relationships presented may be limited to this group only. Expanding the model to other customer segments, product categories, and topics beyond health could provide a more comprehensive understanding of consumer behavior.

We note that, after applying our eligibility criteria – specifically targeting respondents who live in households affected by food intolerances or allergies and are responsible for household food purchases – only 37% of those initially willing to participate qualified to complete the survey. This was done deliberately to accurately target the population relevant to the research objectives and to ensure the validity of the data and the meaningfulness of the findings. However, future studies may benefit from examining larger samples or using complementary methods to confirm the representativeness and generalizability across different household structures and consumer responsibilities.

## 6. Conclusion

The escalating prevalence of diet- and lifestyle-related ailments, alongside the growing incidence of allergies and intolerances, has propelled the expansion of the market for specialized foods, devoid of unsuitable components. Despite potential nutritional drawbacks [13], the perceived health benefits associated with these products have bolstered their popularity [12,132]. However, ongoing research on food allergies and intolerances encounters various challenges, underscoring the need for a deeper understanding of the subject matter. In our study, we present a combined model integrating elements from the Theory of Planned Behavior (TPB), Health Belief Model (HBM), and Protection Motivation Theory (PMT) to comprehensively grasp the factors influencing health behaviors in households affected by food allergies and intolerances.

Our model elucidated that all constructs of the Theory of Planned Behavior (perceived behavioral control, attitude, subjective norm) exhibited the highest regression weights, offering a more comprehensive account of intentions within the resultant model compared to other constructs. Notably, response cost, characterized by their significantly negative regression weight, emerged as a crucial component within the intricate model. It is noteworthy that response cost was the only significant construct retained from the Protection Motivation Theory (PMT) model. Within the framework of the Health Belief Model (HBM), three constructs retained their significance, with health motivation demonstrating a higher regression weight. This integrated model enhances our understanding of consumer intentions and behaviors related to foods suitable for individuals suffering from ARF, offering insights into the complex factors influencing purchasing decisions among households affected by food allergies and intolerances.

## Supporting information

S1 Data Food_intolerance_ACQfilter. (CSV)

S2 Data Food_intolerance_ACQfilter (XLSX)
